# Reflections on epidemiological investigations of sepsis in the Asian Region

**DOI:** 10.1186/s13054-024-04849-8

**Published:** 2024-02-26

**Authors:** Mingqiang Wang, Lin Zhong

**Affiliations:** 1 Department of Critical Care Medicine, Zhengzhou University People’s Hospital, Henan University People’s Hospital, Zhengzhou, China; 2 Henan Key Laboratory for Critical Care Medicine, Zhengzhou Key Laboratory for Critical Care Medicine, Henan Provincial People’s Hospital, Zhengzhou, China; 3 Department of Critical Care Medicine, The First People’s Hospital of Pinghu, Pinghu, China

We read with interest the study by Li et al. [1, 2]. The authors conducted a comprehensive investigation of septic patients in Asian ICUs. Here, we have some concerns and suggestions that we would like to address.

In Asia, especially in low- to middle-income countries or regions, the proportion of patients' families requesting withdrawal of treatment is not negligible. Although this phenomenon is rarely reported, as clinicians in China, We have witnessed numerous instances where patients' families requested to abandon treatment due to economic or other reasons. The act of abandoning treatment not only involves directly discharging patients from the ICU to go home but also encompasses refusing various expensive medications and examinations within the ICU, thereby forgoing opportunities for treatment.

It raises a concern why the authors' comprehensive investigation did not mention any cases of patients facing these circumstances. It seems that all patients in the study did not abandon treatment. We believe that this situation is not exclusive to China and may be even more severe in lower-income countries. In this study, we think that this bias could significantly impact the conclusions drawn from the research.

The authors' two studies are excellent; however, a notable limitation is the lack of understanding of local medical customs, laws, and other contextual factors. Few studies have pointed out the incidence of such situations and their impact on clinical research. In future studies of this nature, it is imperative to comprehensively consider factors such as legal aspects, cultural practices, and the local healthcare landscape to better prepare for more thorough data collection.

## Data Availability

None.
